# Cognitive effects after ECT: how to treat them?

**DOI:** 10.1192/j.eurpsy.2023.99

**Published:** 2023-07-19

**Authors:** E. Verwijk

**Affiliations:** Cognitive effects of electroconvulsive therapy (ECT): where do we go from here?, Amsterdam, Netherlands

## Abstract

**Abstract:**

How far have we progressed in the field of treating cognitive side effects? Six months after the last ECT treatment, about 15% of patients still have such symptoms, limiting daily life. So far, studies in the field of cognitive training for ECT-related cognitive side effects show few positive effects. Nevertheless, these patients seem to help well. Because they have no structural damage to their brain, training with long-term effectiveness may be available for them. Amsterdam University Medical Centers started the CONNECT clinic (Cognitive and Neuropsychological Aftercare ECT) in early 2022 for diagnosis and treatment of patients with long-term cognitive complaints (longer than six months) that hamper their daily functioning. After the intake and neuropsychological diagnostics, treatment starts if there is an indication. Patients are then offered ’cognitive strategy training’ in an individual course. Developed in the field of neuropsychological rehabilitation, these help to get a grip on cognitive complaints by improving memory or executive functioning with learning strategies. Our initial experiences with patients show that patients master these strategies very well and quickly. We present a pilot project from the Amsterdam UMC where an individualized treatment-programme for long-term cognitive complaints after ECT is being implemented and evaluated.

**Disclosure of Interest:**

None Declared

